# Editorial Independence in the Electronic Age: New Threats, Old Owners?

**DOI:** 10.4103/0973-1229.40568

**Published:** 2008

**Authors:** John Hoey

**Affiliations:** * *Queen's University, Kingston Ontario, Canada*

**Keywords:** Editorial Independence, Editorial Freedom, Editorial Integrity, Journal Profitability, Conflict of Interest, Electronic Publication, Pharmaceutical Advertising

## Abstract

Editorial independence is crucial for the intellectual life of a scientific journal. A journal exists only as an idea created by authors and readers, with some editorial orchestration. Editorial independence can be compromised by pressure put on editors by their owners–whether commercial publishers or professional organizations. Both types of owners rely heavily on income from paid advertising in their print journals. Yet, the massive expansion of journal readership that has resulted due to the development of the Web has effected a marked shift in the readership of the journal, both geographically and intellectually, producing a new community of users who see only electronic versions of the journal. Commercial pressures on owners to satisfy the interests of the (mainly national and professional) print readership conflict with the editorial independence needed to respond to the vast Web constituency. This is a major source for compromise of editorial independence. Reduction of commercial pressures by transferring editorial costs to authors and by other cost-reducing models are discussed in this article.

## Introduction

Editorial independence is the indispensable principle governing the intellectual environment that nourishes the soul of a scientific journal. A journal is an idea or set of ideas created by readers and authors, orchestrated to some extent by editors. For this community to function with a high level of efficacy, editors must insist upon the principle of intellectual integrity and defend the rights of authors and readers to speak openly and honestly.

George Orwell, writing in 1945 on the 300^th^ anniversary of the printing of John Milton's *Areopagitica*, particularly noted Milton's arguments that there are two enemies of intellectual freedom: those that are theoretical – essentially various forms of totalitarianism – and those that are practical – monopolies and bureaucracies (Orwell, 1944). Scientific journals (especially general medical journals) face these enemies today, particularly the practical ones.

Most readers or authors of medical journals give little thought to editorial independence – we take our intellectual liberty as a given. Editors, however, often experience threats to their editorial independence. I “became” an editor in 1996 and almost immediately was confronted by Milton's theoretical enemies; later, with the development of the Web, I became acquainted with the practical ones. A combination of both brought an end to my editorship of *CMAJ* (Shuchman and Redelmeier, 2006).

So what is editorial independence in a medical journal? The International Committee of Medical Journal Editors (ICMJE) statement on editorial freedom, developed by Robert Fletcher for the World Association of Medical Editors (WAME) and later adopted by the ICMJE, says:

*“…editors-in-chief should have full authority over the editorial content of their journal. Journal owners should not interfere in the evaluation, selection, or editing of individual articles either directly or by creating an environment that strongly influences decisions. Editors should base decisions on the validity of the work and its importance to the journal's readers, not on the commercial success of the journal. Editors should be free to express critical but responsible views about all aspects of medicine without fear of retribution, even if these views might conflict with the commercial goals of the publisher.” From ICMJE statement on editorial freedom (www.icmje.org; ICMJE, 2006)*.

Parsed, this means: “The editor is responsible solely for the content of the journal. The owner is responsible solely for hiring and firing the editor.” The owner cannot even “create an environment that strongly influences (editorial) decisions.” There is no provision for common ground on which to hold a discussion about editorial direction and even less about particular manuscripts. When the American Medical Association (AMA) fired George Lundberg, then editor of *JAMA*, for publishing a scientific article which the Association found offensive to its national political agenda at the time (Hoey *et al*., 1999), the AMA did not negotiate with Lundberg. He published. They fired.

## Theoretical Censorship

The theoretical infringements of editorial independence, at least some of the most spectacular flameouts of editors and owners, including probably my own (Editorial, 2005), are repeats of previously described dust-ups. Many undoubtedly happen because the resolution of a dispute over editorial content inevitably boils down to the owner sacking the editor, hiring another, and hoping for the best.

Often these theoretical disagreements occur because the extent of editorial freedom, though broad, is not limitless. Some limits are imposed by the nature of the journal - a medical journal normally would not publish an article on theoretical physics. Other limits reflect the historical or traditional boundaries of a journal, which define the scope of its editorial prerogative. For example, *The Lancet* has a long history of tackling the broader social determinants of human health, a tradition that has been maintained from founding editor Thomas Wakley's editorials condemning polluting factories in Dickensian London (1855) to current editor Richard Horton's editorial plea to the publisher, Elsevier, to divest itself of its arms-trade business (Editorial, 2005). On the other hand, many eminent general medical journals have a tradition of sticking close to the practice of bedside medicine by physicians.

Journals published by professional societies such as the Canadian, British, or American medical associations may censor editors who publish articles critical of physicians or those that are perceived to threaten their core goals and objectives; professional associations of physicians are lobby groups that exist to promote the interests of their members. My sacking and Lundberg's, could be viewed (with considerable accuracy) through the lens of theoretic censorship.

## Practical Censorship of Editorial Independence

But in the day-to-day work of editing and publishing there is a more subtle infringement of editorial independence, which has nothing to do with political or moral perspectives; it comes from the commercial interests of the owners, the second of Orwell's enemies. The ICMJE/WAME statement could not have put it with greater clarity:

“Editors should base decisions on the validity of the work and its importance to the journal's readers, not on the commercial success of the journal.”

I suspect this ICMJE admonition or guidance is included to define a clear boundary in a narrow area – that pharmaceutical company support of a medical or health sciences journal should come with no strings attached. For example, the editors of, say, the *Annals of Internal Medicine* should not be influenced in their decisions about a manuscript that describes a patent-protected drug by the knowledge that if they accept it, the publisher (the American College of Physicians) would pocket very large sums from the pharmaceutical company for article reprints. Nor should the owners seek advertisements from commercial companies and charge extra fees to have them placed adjacent to favourable articles about their products. Nor, indeed, should paid advertising in a journal be accepted when it is dressed up as peer-reviewed content (advertorials).

There is, however, a more insidious “practical” censorship that revolves around the commercial success of the journal. Editors are profoundly interested – or they should be – in the profitability of their publication. It costs money to run a journal. At a minimum, the editor's interest must extend to achieving the break-even point between revenue and expenses. For owners, any profit from their journal is not only desirable but essential (especially so for commercial companies like Elsevier). And many association-owned journals generate profits for their owners – profits that the owners expect, and use, to further their lobbying interests.

Practical censorship of editors will grow in importance as the costs of running a journal increase and, I will argue, because of the shifting readership resulting from the Web. Because the practical commercial forces are so different, it will be useful to distinguish between journals with high impact factors (say the top four or five in a category such as general and internal medicine) and those with modest or low impact.

Those with high impact have multiple revenue streams, including substantial individual and library subscriptions, reprint sales, and commercial and classified advertising. They are exclusively closed-access journals in that much of their content (and often all) is limited to paying subscribers. (I will ignore current Orwellian terminology that allows many of these closed sites to call themselves “open.”) Editors of these publications must keep their subscribers in sharp focus and respond to their needs with regard to the journal's contents. If they are successful then the practical constraints on editorial independence disappear into the intellectual community of authors and readers, which can grow and adapt as the interests of this community change over time and space. *The Lancet*, for example, under the current editorial team has rather successfully, it appears, shifted its reader and author community into a global enterprise, presumably maintaining its subscriber bases and we hope expanding them. (Of course, at the cost of excluding a much larger intellectual community that cannot afford the subscription fees.)

Those with lower impact have subscription bases that are usually limited to sponsoring society members [*CMAJ* is a good example] where the “subscribers” are in fact receiving the journal at no or little cost because they are members of their professional association. Because these journals rarely publish top-tier research, the income from reprint sales is low or absent. Journal revenue is almost entirely dependent on commercial and classified employment advertising.

Advertisers are willing to pay for “reader eyeballs,” hoping that their advertisements will be seen and prescriptions written or jobs filled. This business model works well if costs are held in check and the print readership maintained. However costs cannot be restrained, especially in a very competitive editorial environment, with increasing numbers of journals vying for the same authors. In addition, the captive association membership is stagnant at best and competition for reader eyeballs is increasing. *CMAJ* for example competed for media buyers’ attention with travel, humour and pseudo-CME (continuing medical education) journals. Further, the reader eyeballs, even of the captive membership of professional associations, are drifting to the Web and e-versions of their own and others’ journals, away from revenue-generating print versions.

Secondly, and of much greater importance, the readership and authorship of even low-impact journals has been profoundly influenced by the Web. The Web was commercially born about a decade ago. In 1997, the US Library of Medicine put its entire *Medline* collection on the Web (as PubMed) and made access entirely free to users. A year later, Richard Smith, then editor of *BMJ*, boldly put the entire contents of the *BMJ* on the Web with the result that in 2007, even after the *BMJ* closed its site to non-subscribing visitors, it had at its most recent survey 1,216,000 unique users, while the print version circulation was not much over 125,000. Seeing this success almost all other journals followed the *BMJ*'s example.

These were heady times, and merry too. Manuscript submissions, even to lower-impact journals available on the Web, increased dramatically. So did letters to the editor. The community of users (readers and authors) of all journals became larger and much more diverse than the former national or specialty-society readerships, extending to the public as well as to the media, who were able to more easily access content.

This inevitably led to conflict between editors and owners. Because pharmaceutical and classified advertising revenue is virtually nonexistent for Web versions of a journal (for many reasons, but mainly because advertisers target audiences within national boundaries), owners of these journals are primarily and necessarily interested in satisfying the readers of their print journals.* Their editors, on the other hand, are increasingly interested in, and indeed following and trying to catch up with, their growing global intellectual communities of readers and authors.

Thus, owners want more drive-through tidbits that need less science, less editing, more bullet points, charts and graphs, and if possible travel advice and cartoons, that will deliver national prescribing-physician eyeballs to the attention of media buyers; at the same time, editors are pursuing the more sophisticated, and generally younger, new readership that is interested in more global problems (defined as those that cross national borders). Editors with large costs to cover just to break even or to deliver the profits that owners have grown accustomed to getting (in order to continue the lobbying efforts of the professional society), must curtail their own editorial independence in the chase for money.

This influence on, and curtailment of, editorial scope is subtle but of great import. Journals must grow, they must change, they must follow both the clinical science of medicine as it extends far beyond the hospital or clinic *and* their growing international constituency of readers and authors. For low-impact journals it is unlikely that revenue from the Web will keep pace with costs. Thus, journals will die or editors, in the chase for advertising revenue from print readerships, will have their independence severely curtailed. But perhaps there are other options to intellectual bankruptcy?

## Revenue Generation Through Author Fees and Reduced Editorial Costs

The Web, while paradoxically crippling low-impact journals, creates opportunities for journals with different diffusion models. When former editors and board members of *CMAJ* created *Open Medicine* (www.openmedicine.ca), they used the free journal publishing software of the Public Knowledge Project led by John Willinsky (http://pkp.sfu.ca/). Their Open Journal Systems now house over 900 journals in 10 different languages. This model immediately avoids about half of the standard journal costs, mainly those of paper, printing, and mailing of the journal. And, by way of the operating grants of the Open Knowledge Project, eliminates most of the electronic and server storage costs.

There remain, however, even for many low-impact journals, substantial editorial costs (salary of editor(s), copyeditors, technical editors, and so on). There are three, and possibly four, approaches to the need for unbiased sources of revenue for Web-only journals. I will ignore access fees which, for very high-impact factor journals, may provide a sufficient subscription revenue stream. For almost all other web-only journals, however, few readers appear willing to buy subscriptions for online access or to pay the very high fees currently charged for a single article download. Access fees also are inversely correlated to online readership volume and thus will curtail both editorial reach and any commercial advertising revenue that might one day be derived by the combination of huge readerships and miniscule download charges.

### 1. Author Fees

The BMC journals and the Public Library of Science (PLoS) journals are examples of revenue generation through author fees. Expenses incurred are mainly to pay the editorial team, the technical editors, and support staff and to cover the costs of online publishing (avoiding entirely the costs of paper and postage because there is no print version). Nonetheless, author fees are substantial. At *PLoS Medicine*, charges to authors for an accepted paper are USD $2,750. But even if all authors paid this fee (and they do not) the fee is too low to cover costs. The costs of editing just one paper at the *Annals of Internal Medicine*, a top medical journal, are USD $13,000 (Editorial, 2007). The downside of author charges are evident and very likely their presence, even with a possible fee waiver for authors unable to pay, reduces the number of submissions and biases the scope of submissions towards areas of research and scholarship that are well funded.

### 2. Lower Editorial Costs

A second alternative to alleviate financial pressure on editorial freedom is to reduce editorial costs. Here there are two journals worth examining. *PLoSONE*, the newest journal incubated by the PLoS, is designed to publish high-quality research, opinion, and commentary at a low cost, entirely paid for by smaller author levies for accepted articles, which is currently set at USD $1,250. This model uses a large number of volunteer editors who receive submitted articles and can decide to peer review them or not, to request revised manuscripts, and to make a decision on publication. The accepted article is then returned to the authors. *PLoSONE* does not copyedit articles but rather urges authors to have them professionally copyedited (at the author's expense – usual cost about USD $250 to $350 – and prepared for electronic release in a version that meets PLoS standards; a list of PLoS recommended companies who provide this service is available).

Backing up this abbreviated prepublication peer and editorial review at *PLoSONE* is a serious attempt to facilitate postpublication peer review through reader comments that are inserted directly into the published article; discussion groups that can be generated by readers; and a reader rating system across several categories, each with Lickert stars (similar to video ratings on YouTube). Thus, over time, articles should emerge that have considered rankings by readers. Further, one can examine article citations – a measure of the importance of the article – with Google Scholar's free citation database.

The *PLoSONE* editors and the large volunteer stable of contributing or corresponding editors thus escape – except for the almost modest author charges – the economic limitations of their editorial freedom. They can publish anything they like as long as authors are willing to submit and can make (collectively) the payments. (Charges are based on ability to pay.)

### 3. Editorial Communities

Another way to reduce commercial pressures on editorial independence is to virtually remove direct editorial costs by relying on communities of interested volunteers. *Open Medicine* might be an example. *Open Medicine* is anchored on two principles: 1) That because pharmaceutical advertising places severe limits on editorial freedom, it must not be used to finance journal operations; and 2) that medical journals of high quality must be open and free to all end users, whatever their economic resources – that science and medicine are part of the commons (Maskalyk, 2007).

The founding editors and board members – mostly former members of *CMAJ* – who had experienced what they rightly interpreted as theoretical (totalitarian) and economic (practical) infringements of their editorial independence by the CMA, sought a platform for editorial independence that was ideologically and economically democratic – similar to Jim Wales’ Wikipedia. (www.wikipedia.org). (A good video of Wales explaining the rationale for Wikipedia and how it functions is available as a lecture he delivered at Stanford University in 2005 at http://ia300106.us.archive.org/3/items/HowardRheingoldIFTFStanfordHumanitiesLabJimmyWales/wales.mov.)

In line with these principles, and following to some extent the Wikipedia model, *Open Medicine* is a volunteer organization of editors, copyeditors, technical support persons, media advisors, and a wide range of other talent that aims to publish high-quality research, commentary, and opinion on any area of health and health care, the scope of which will be determined by contributors, readers, and in fact anyone who wants to participate. The scope of editorial freedom of this new journal will thus be determined by the broader constituency, one that my colleagues and I once described as a “constituent assembly” (Hoey *et al*., 1999).

### 4. Self-publication: A Viable Option?

Is there a forth channel or option to PLoS and BMC journals, to *PLoSONE*, or to *Open Medicine*? Perhaps. Further along the spectrum of editorial independence is self-publication. It will take time to see if this activity can develop momentum and critical mass. It will require courageous, productive, and highly regarded authors to reject traditional publishing models and self-publish. There is no reason why sites could not be developed for open prepublication, peer review, and comment, along with opportunities, such as those currently in *PLoSONE*, for post-publication peer review and rating – without the editors.

## Concluding Remarks

This view of the future is utopian and undoubtedly simplistic. Many will argue that cooperative intellectual enterprises are unsustainable; others, that they bring just another form of editorial constraint, a sort of intellectual totalitarianism. Still others foresee a Web that is pay-per-view, with very low per-view costs offset by high volume. Nonetheless, I am encouraged by the 900 journals using the free software of the Open Knowledge Project, by the success of the BMC and PLoS journals, by the experiments of PLoSONE and *Open Medicine*, and by the rapidly expanding communities of bloggers, podcasters, open software developers, and Wikipedia-like contributors that populate the Web. Perhaps we do have the intellectual energy to make free scientific publication work.

By moving editors and editorial functions firmly into the public space we will achieve the greatest and least encumbered forms of editorial freedom and independence.

In the movement along these lines I expect we will see survive, in a more traditional editing and publishing mode, only a few excellent and very high impact factor journals. They will be valuable for those who want (and are able to pay for) high-quality and quantity editors to do the work of choosing what is worth reading and editing it so that it is readable. Other journals, labouring under the increasingly expensive economics of their publishing models will be obliged to chase an increasingly narrow readership that will remain country specific in a world that has become country nonspecific in everything; except, unfortunately, in nationalism.

### Take Home Message

There are spectacular infringements of editorial independence such as the firing of the editor of *JAMA* by the AMA over the publication of a particular article or my own sacking by the CMA. These are examples of differences in outlook or worldview between owners (publishers) and editors. A much more common infringement of editorial independence, affecting almost all editors, is editorial interference from commercial pressures to make money to cover journal costs and/or generate profits for the publishers. As most revenue for medical journals comes from paid advertising in print subscriptions, the commercial interference with an editor's freedom is increasing as print readership stagnates or declines and electronic readership, which does not generate revenue, explodes.

## Questions That This Paper Raises

1. What are the main threats to editorial independence of general medical journals?
2. Is editorial integrity compromised by the need for the journal to make money?
3. Could these commercial pressures on editorial independence be lessened by reducing editorial costs and oversight?
4. Could editors reduce their editorial and production costs and thus lessen commercial pressures to chase a progressively smaller and smaller print readership?

## About the Author

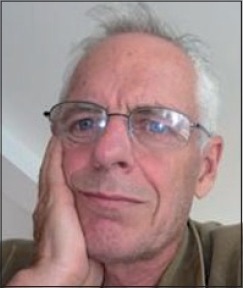



John Hoey, an internist and public health physician, was editor of CMAJ from 1996 to 2006. He is currently on the faculty at Queen's University, Canada where he is involved in developing public health resources and teaching. His current research interests include a study of conflicts of interest in contract research grants between the private sector and universities in Canada (at the University of Toronto), research reporting guidelines, and the development of guidelines for reporting of research (the EQUATOR group, Oxford) and, most recently, in the area of the use of vitamin C (ascorbic acid) for chemotherapy of advanced malignancies (with colleagues at NIH and at McGill).

## Footnotes

* Online advertising may develop a viable and substantial commercial advertising revenue stream for a few very high-impact factor journals with many millions of “readers,” but not for the 90% of journals that, while increasing their communities of readers, will never see them grow to revenue-generating sizes.
